# Clinical value of metagenomic next-generation sequencing by Illumina and Nanopore for the detection of pathogens in bronchoalveolar lavage fluid in suspected community-acquired pneumonia patients

**DOI:** 10.3389/fcimb.2022.1021320

**Published:** 2022-09-27

**Authors:** Jing Zhang, Lin Gao, Chi Zhu, Jiajia Jin, Chao Song, Hang Dong, Zhenzhong Li, Zheng Wang, Yubao Chen, Zhenhua Yang, Yan Tan, Li Wang

**Affiliations:** ^1^ Department of Respiratory and Critical Care Medicine, Nanjing First Hospital, Nanjing Medical University, Nanjing, China; ^2^ State Key Laboratory of Translational Medicine and Innovative Drug Development, Jiangsu Simcere Diagnostics Co., Ltd., Nanjing, China

**Keywords:** metagenomic next-generation sequencing, bronchoalveolar lavage fluid, community-acquired pneumonia, Illumina, nanopore, diagnostic value, pathogenic identification

## Abstract

At present, metagenomic next-generation sequencing (mNGS) based on Illumina platform has been widely reported for pathogen detection. There are few studies on the diagnosis of major pathogens and treatment regulation using mNGS based on Illumina versus Nanopore. We aim to evaluate the clinical value of metagenomic next-generation sequencing (mNGS) by Illumina and Nanopore for the detection of pathogens in bronchoalveolar lavage fluid (BALF) in suspected community-acquired pneumonia (CAP) patients. BALF samples collected from 66 suspected CAP patients within 48 hours of hospitalization were divided into two parts, one for conventional culture and the other for mNGS by two platforms (Illumina and Nanopore). The clinical value based on infection diagnosis, diagnostic performance for main pathogens and treatment guidance were assessed. More types of species were detected by Nanopore than Illumina, especially in viruses, fungus and mycobacterium. Illumina and Nanopore showed similar detectability in bacterium except for mycobacterium tuberculosis complex/nontuberculosis mycobacteria. Pathogenic infection was established or excluded in 53 of 66 patients. There was little difference in the coincidence rate between Illumina and Nanopore with the clinical diagnosis, but both were superior to the culture (57.81%, 59.38%, 25%, respectively). Compared with Illumina, the diagnostic area under the curve of Nanopore was higher in fungi, but lower in bacteria and Chlamydia psittaci. There was no statistically significant difference between Illumina and Nanopore in guiding drug treatment (56.1% vs. 50%, *p*=0.43), but both were superior to the culture (56.1% vs. 28.8%, *p*=0.01; 50% vs. 28.8%, *p*=0.01). Single inflammatory indicators could not be used to determine whether the patients with culture-negative BALF were established or excluded from infection. The species detected at 1 h and 4 h by Nanopore were consistent to some extent, and its turn-around time (TAT) was significantly shorter than Illumina (*p*<0.01). Illumina and Nanopore both have its own advantages in pathogenic diagnosis and play similar roles in infection diagnosis and guiding clinical treatment. Nanopore has a relatively short TAT, which may be promising in rapid etiological diagnosis of acute and critically ill patients.

## Introduction

Lung infection is one of the major causes of death worldwide (Magill et al., 2014). In terms of mortality, pneumonia caused 3.2 million deaths worldwide, surpassing all other infectious diseases including tuberculosis, human immunodeficiency virus (HIV) and malaria and ranking the fourth among the top 10 causes of death globally in 2019 (WHO, 2020). Lung infection is also one of the most serious public health problems in China. A study including 16,585 patients with community-acquired pneumonia (CAP) in China showed that the number of patients aged 5 years and below (37.3%) and over 65 years (28.7%) were significantly higher than that of patients aged 26-45 years (9.2%) (Liu et al., 2013). However, due to pathogenic diversity (bacteria, fungus, viruses, atypical pathogens and parasites) in pneumonia and infectious complexity (Renaud and Campbell, 2011; Ruppé et al., 2016; De La Cruz and Silveira, 2017), accurate and timely diagnosis of pathogens is difficult but vital for proper treatment of pulmonary infection and for improving the prognosis, especially for multi-drug treatment of patients with infectious disease.

Currently, conventional methods for diagnosing the cause of infection include microbial culture, serology, and polymerase chain reaction (PCR)-based nucleic acid tests (Loeffelholz and Chonmaitree, 2010; Labelle et al., 2010). However, the diagnostic efficiency of these methods is affected by a long time, limited detection range, low positive rates, and small flux (Huffnagle et al., 2017). For example, the long turn-around time (TAT) of microbial culture cannot detect viruses and parasites, and the sensitivity of antigen/antibody detection may be limited (Loeffelholz and Chonmaitree, 2010). Although traditional PCR-based nucleic acid tests are highly sensitive and specific, they detect a limited range of microorganisms and may not include the pathogen causing the infection. Recently, metagenomic next-generation sequencing (mNGS) based on Illumina has become one of the potential technologies for etiological diagnosis, and has been widely used in clinical detection of infectious pathogens, especially rare or emerging pathogens (Hilton et al., 2016; Salzberg et al., 2016; Somasekar et al., 2017; Lu et al., 2020). mNGS was found more sensitive and specific than microbial cultures, especially for the detection of Mycobacterium tuberculosis (MTB), viruses, anaerobic bacterium, and fungus (Forbes et al., 2017; Miao et al., 2018; Zhang et al., 2019), and its performance was less affected by prior antibiotic exposure (Zhou et al., 2019).

With the development of sequencing technologies, Nanopore sequencing (MinION sequencer by Oxford Nanopore Technologies), converts the current signal difference generated when nucleic acid molecules pass through nanopores into the bond sequence of chemical signals, realizing the direct reading and sequencing of nucleic acid molecules, and getting rid of the amplification process of PCR. The bias and introduced errors caused by PCR amplification are avoided. Meanwhile, Nanopore sequencing has a longer read length, up to 2Mb, which greatly improves the accuracy of the comparison of near pathogenic microorganisms. In addition, the characteristics of real-time analysis further improve the overall test cycle. Nanopore and Illumina are both reported in the detection of main pathogens, with a consistency of more than 60%. Illumina gave feedback within 3 days, while Nanopore gave feedback within 1 day. The overall sequencing process of Nanopore was optimized, with the detection cycle of 6 h (Gu et al., 2021), which met the clinical demand for rapid detection of pathogens in acute and critically ill patients to a greater extent. However, there are few studies on the diagnosis of major pathogens and treatment regulation using mNGS based on Illumina versus Nanopore.

In this study, we applied an optimized detection scheme for clinical respiratory pathogens based on Nanopore sequencing to identify the pathogens in suspected CAP, and compared the pathogenic detectability of Nanopore sequencing with Illumina and traditional culture methods. Additionally, infection diagnosis, diagnostic performance for main pathogens and treatment guidance were also assessed.

## Materials and methods

### Study population and design

A prospective analysis of suspected CAP patients was performed at Department of Respiratory and Critical Care Medicine in Nanjing First Hospital from October 2021 to April 2022. The diagnostic criteria of CAP were in accordance with Chinese guidelines for CAP in adults (Cao et al., 2018). The samples of bronchoalveolar lavage fluid (BALF) collected within 48 h of hospitalization were divided into two parts, one for conventional culture and the other for mNGS by two platforms (Illumina and Nanopore). The inclusion criteria were as follows: (i) sufficient BALF collected for laboratory testing within 48 h of admission; (ii) informed consent from the patients themselves or surrogates to the study; (iii) complete clinicopathological and follow-up information. Patients who refused to sign informed consent were excluded from this study. Ethical approval was achieved from the hospital’s ethical committee (NO. KF20220516-05), and informed consent was obtained from each patient before bronchoscopy.

### Evaluation on infection diagnosis, diagnostic performance for main pathogens and treatment guidance

The infection diagnosed was classified into 5 types: established, excluded, suspected, unknown causes, and other non-infectious factors. To evaluate methodological efficiency of infection diagnosis, we compared the results of different methods to check whether they covered the infection diagnosis or were identical. The pathogens were mainly classified into bacterial, MTB/non-tuberculous mycobacteria (NTM), fungal, viral, atypical, and mixed types, which were determined by the chief physician in conjunction with other tests. With the clinical etiological diagnosis as the golden standard, the diagnostic performance for major pathogens by the Illumina, Nanopore and culture methods were compared. Regarding the treatment, continuous experiential medication, targeted drug therapy, reversed drugs and hospital transfer were categorized. To evaluate the potential for treatment guidance, we retrospectively analyzed whether the treatment should be adjusted based on the detection results of the three methods.

### DNA extraction

First, transfer 1-2 mL of BALF sample to a clean centrifuge tube of 2 mL and centrifuge at 14,000 g for 5 min. Second, carefully aspirate the supernatant, and keep 200 µL of the supernatant and pellet in a centrifuge tube for later use. Third, add 10 µL of the prepared lysozyme (20 mg/mL) and incubate at 37°C for 15 min, then put in a MP Lysing Matri E tube and add 200 µL of GB lysis solution. Next, shake the MP tube on a wall breaker (FastPrep-24™ 5G) at 6 m/s for 120 s, and centrifuge at 14,000 g at low temperature for 5 min. After centrifugation, take all the supernatant and add it to a new EP tube of 2mL. Finally, use a micro-sample genomic DNA extraction kit (DP316, Tiangen) to extract the nucleic acid.

### Library preparation and sequencing

NEBNext Ultra II DNA Library Prep Kit (New England Biolabs Inc.) was used to construct Illumina sequencing libraries and Nextseq 550 DX (75 bp single-end reads; Illumina) was used for sequencing. About 20 million reads were generated for each sample. The Rapid Barcoding Kit SQK-RPB004 (DNA concentration <20 ng/mL; Oxford Nanopore) and SQK-RBK004 (DNA concentration >20 ng/mL; Oxford Nanopore) were used to construct Nanopore sequencing libraries according to the manufacturer’s instructions, and GridION X5 (Oxford Nanopore) was used for sequencing. About 0.8 G of data were generated for each sample.

### Bioinformatics analyses

High-quality sequencing data were generated by removing low-quality reads, including adapter contamination, duplicated reads and short reads (Illumina: length < 50 bp; Nanopore: length <500 bp). An alignment tool (Burrows-Wheeler Alignment) was used to map to a human reference genome (GRCh38) to exclude human sequence data. The remaining sequencing data were aligned to NCBI nt database by SNAP. The mapped data were processed for advanced data analysis with in-house scripts, including taxonomy annotation, genome coverage/depth calculation and abundance calculation. Details were described in our previous work (Guo et al., 2021).

### Statistical analysis

SPSS 22.0 statistical software was used for data analysis, and Graphpad Prism 8 and R were used for plotting. Non-normally distributed data were expressed as the median [first quartile (Q1), third quartile (Q3)], and non-parametric Mann-Whitney U test was used for comparison between groups. The counting data were expressed as the number of cases (percentage) [n (%)], and the data between groups were compared by chi-square test or Fisher’s exact test. We used clinical etiological diagnosis as the reference standard to evaluate the diagnostic efficacy of three methods. 2 × 2 contingency tables and receiver operating characteristic (ROC) curves were used to evaluate the diagnostic efficacy. A two-tailed value of *p*<0.05 represented significant differences.

## Results

### Demographic characteristics of study population

Between October 2021 and April 2022, a total of 66 patients with suspected CAP were enrolled in this study, including 42 males and 24 females. The median age of patients was 68 (58, 72) years. Among 66 cases, 2 died due to tumor progression, 7 were transferred to a designated hospital for treatment due to mycobacterium detection, 1 with unknown pathogens was transferred to other hospital for treatment, and the symptoms of the remaining patients were all improved.

### Genus distribution and consistency of microorganisms detected by Illumina, Nanopore and culture

**Figure 1:**
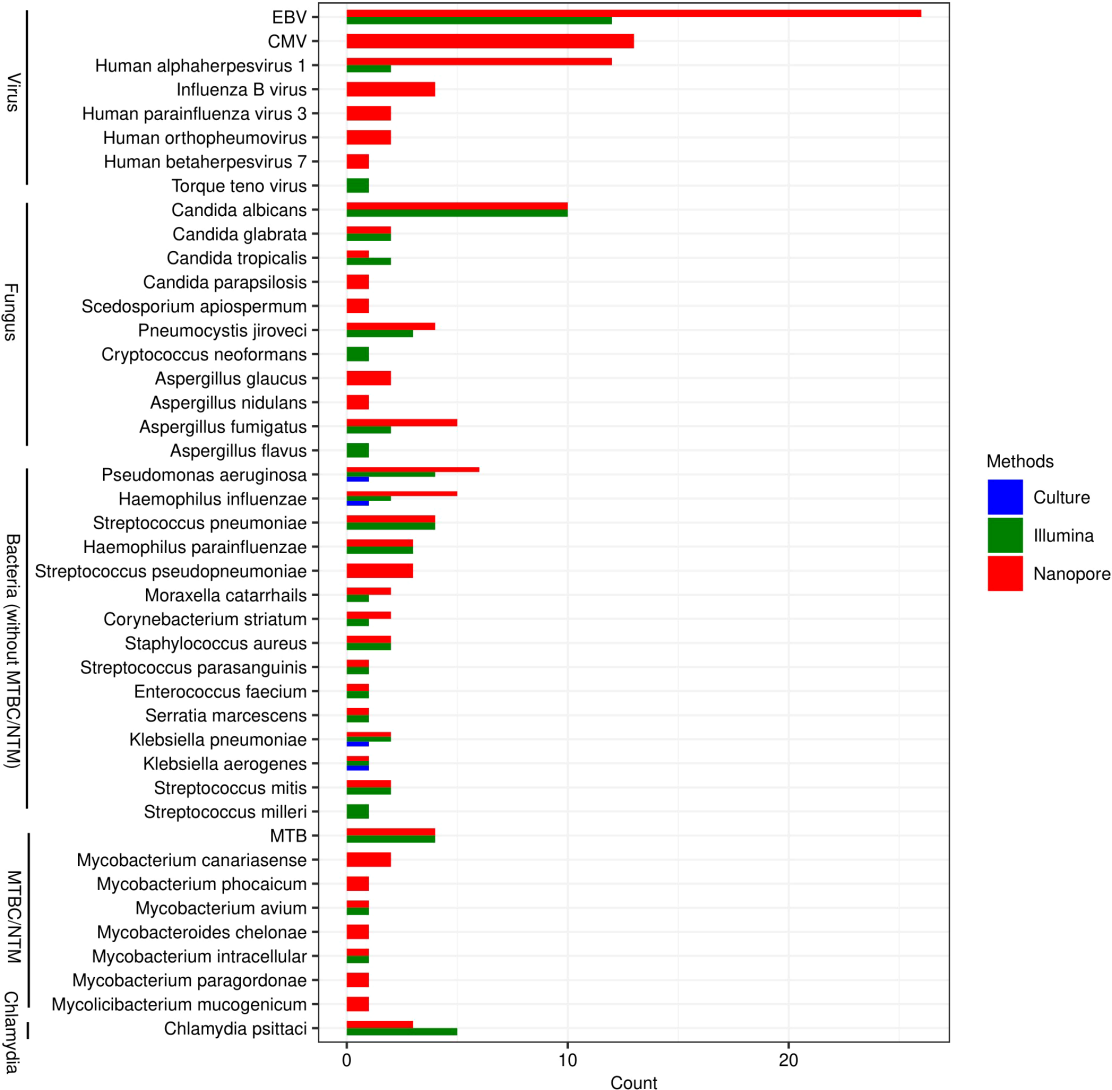
Genus distribution of bacterium, fungus, viruses and chlamydiae detected by Illumina, Nanopore and culture. EBV, Epstein-Barr virus; CMV, Human cytomegalovirus; MTB, Mycobacterium tuberculosis; NTM, Non-tuberculous mycobacteria.

All species that may be pathogens detected by Nanopore and Illumina are listed in **Figure 1**. As it shown, the types of species detected by Nanopore were more than those by Illumina, especially in the detection of viruses (6 versus 3), fungus (9 versus 7) and mycobacteria (8 versus 3). The pathogens in 4 cases of bacterial infection detected by traditional culture method were *Pseudomonas Aeruginosa*, *Haemophilus Influenzae*, *Klebsiella pneumoniae* and *Klebsiella aerogenes*, which were also confirmed by Nanopore and Illumina. In another 4 patients, however, MTB was identified by Nanopore and Illumina, but not by culture. More types of NTM were identified by Nanopore compared with Illumina. *Candida Albicans* was the most commonly detected fungal species, which was identified in 10 cases by Nanopore and Illumina. The most frequently detected viruses were *EBV*, *CMV* and *Human alphaherpesvirus 1*, while only three types of viruses including *EBV*, *Human Alphaherpesvirus 1* and *Torque Teno virus* were detected by Illumina.

**Figure 2:**
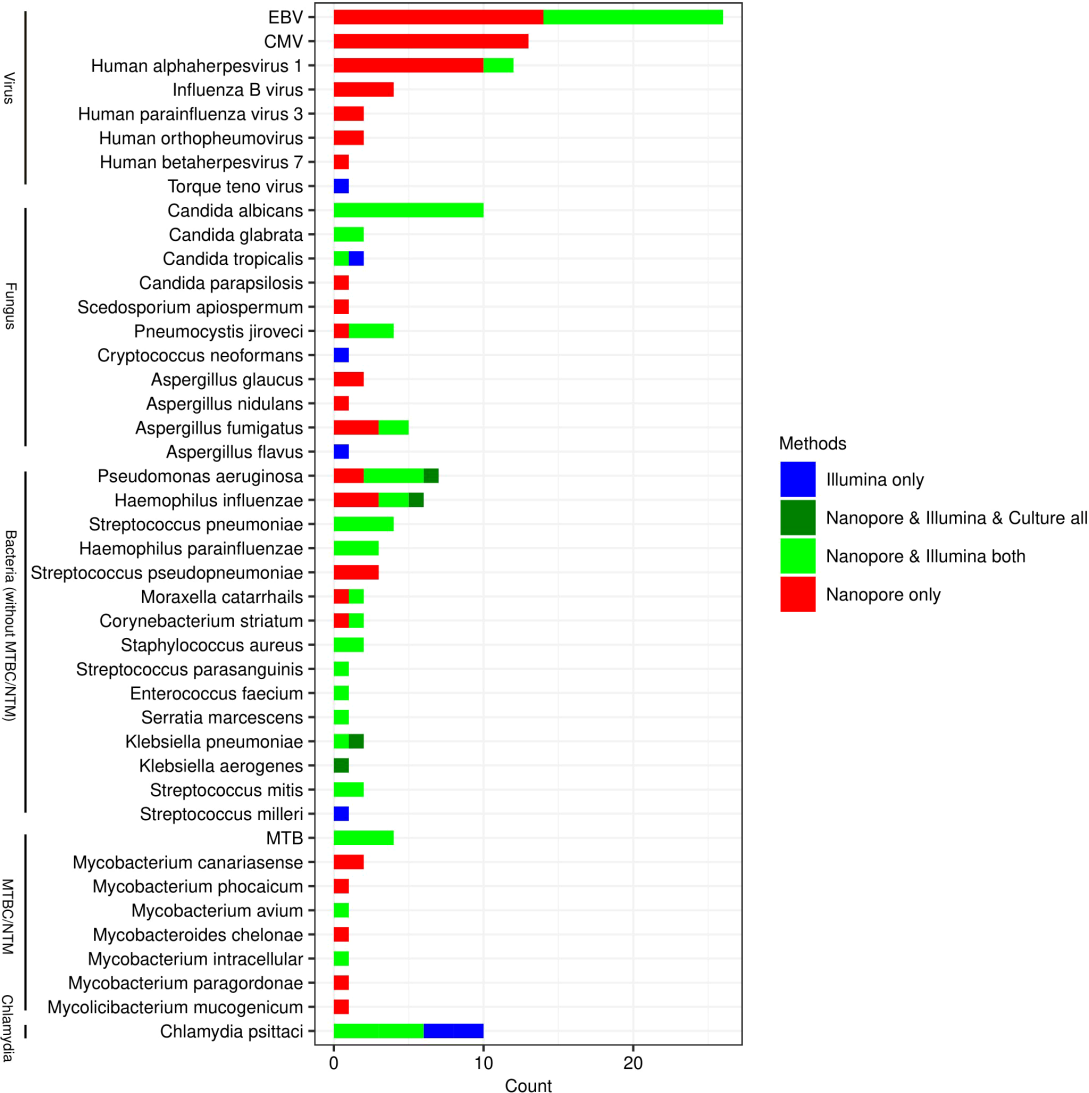
The consistency of pathogens detected by Illumina, Nanopore and culture.

As shown in **Figure 2**, Illumina and Nanopore showed similar detectability in bacterium except for MTBC/NTM. The results of *Klebsiella aerogenes* detected by the three methods were consistent. In this study, as a possible pathogen, *Streptococcus pseudopneumoniae* was only detected by Nanopore, while *Streptococcus milleri* was only detected by Illumina. *Mycobacterium avium* and *Mycobacterium intracellular* were both detected by Nanopore and Illumina in NTM. However, Nanopore also detected other types of NTM. *Candida albicans* and *Candida glabrata* were both consistently detected by Illumina and Nanopore. Illumina only detected 1 case of *Aspergillus flavus* and *Cryptococcus neoformans*. *Candida parapsilosis*, *Scedosporium apiospermum*, *Aspergillus glaucus* and *Aspergillus nidulans* were only detected by Nanopore. In terms of viral detection, *EBV* and *Human Alphaherpesvirus 1* were both partly detected by Illumina and Nanopore, and the rest were only detected by Nanopore. Besides, 1 case of *Torque teno virus* was detected only by Illumina. Three cases of *Chlamydia psittaci* were uniformly detected by Nanopore and Illumina, while 2 cases were detected only by Illumina.

### Distribution of infection diagnosis

**Figure 3:**
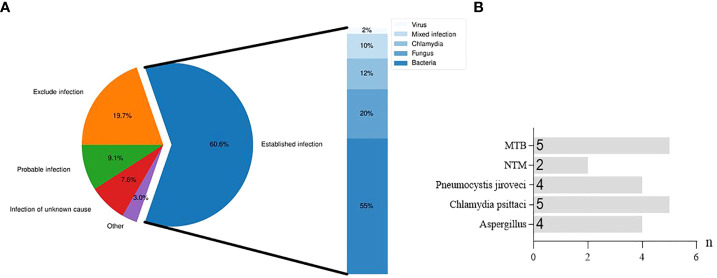
Distribution of infection diagnosis in suspected CAP patients. **(A)**, Diagnostic distribution and etiological classification of infection established in patients with suspected CAP. **(B)**, Specific pathogens detected in bronchial alveolar lavage fluid from patients with established infection.

Distribution of infection diagnosis is described in **Figure 3A**. Among the 66 patients with suspected CAP, 40 cases were confirmed to be infected with pathogens, 13 were excluded for infection, 5 were identified with infection of unknown causes, 6 had suspected infection, and 2 were diagnosed as infection caused by other non-infectious factors. Among 40 cases of definitely diagnosed infection, several specific pathogens were found, such as MTB, NTM, *Pneumocystis jiroveci*, *Aspergillus* and *Chlamydia psittaci* (**Figure 3B**).

**Figure 4:**
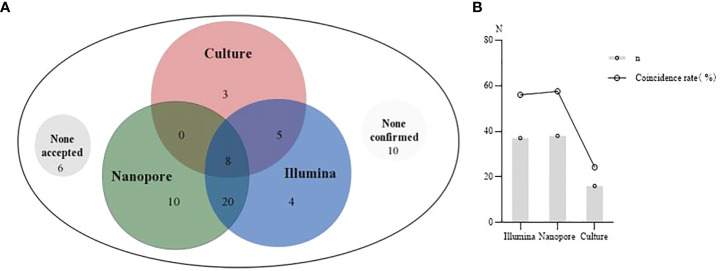
The clinical diagnosis based on Illumina, Nanopore and culture. **(A)**, A Venn diagram of diagnosis based on the three methods. None accepted, none results of the three methods were accepted by clinicians. None confirmed, none results of the three methods were consistent with clinical diagnosis. **(B)**, Comparison of the coincidence rate between the three methods with the clinical etiological diagnosis.

The consistency of Illumina, Nanopore and culture methods with clinical diagnosis is shown in **Figure 4**. In 6 cases, none of the diagnostic results through the three methods were accepted by the clinician. In 10 cases, none of the diagnostic results by the three methods were consistent with the clinical diagnosis (**Figure 4A**). The coincidence rates between the three methods (Illumina, Nanopore and culture) and clinical etiological diagnosis were 56.1%, 57.6% and 24.2%, respectively (**Figure 4B**).

### Diagnostic performance of the three methods for main pathogens

**Figure 5:**
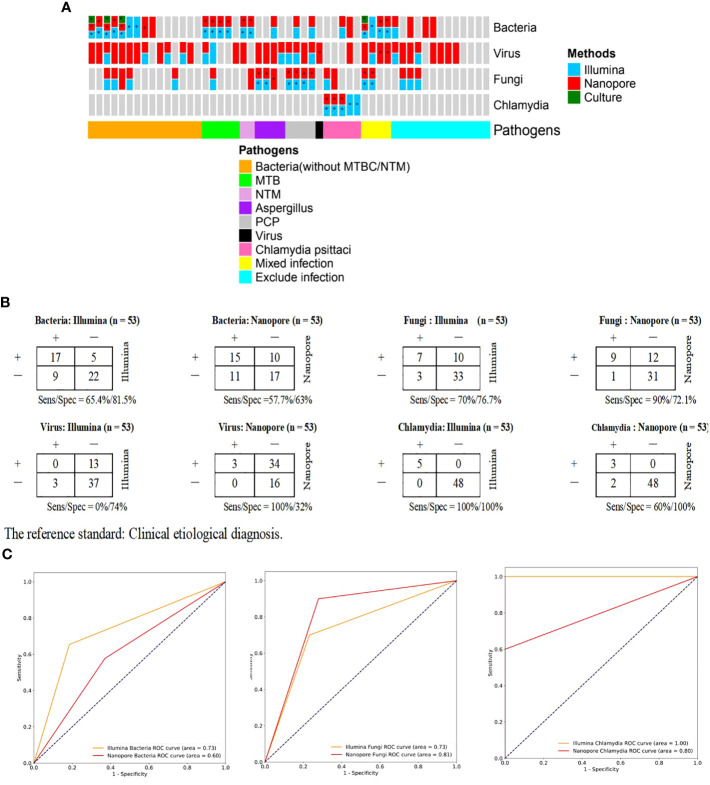
Comparison of diagnostic performance of Illumina and Nanopore for the main pathogens in BALF of patients with suspected CAP. **(A)**, Confirmation/exclusion of pathogenic infection in 53 patients using Illumina, Nanopore and culture. *: The results were consistent with the clinical etiological diagnosis. **(B)**, Contingency tables for the Illumina and Nanopore in detecting different classes of pathogenic microorganisms. Clinical etiological diagnosis was used as a reference method. **(C)**. ROC curves stratified by classes of microbes.

Of the patients with confirmed pathogenic infections (n=40), the pathogens infected were classified into bacterial (without TB/NTM, n=15), *tuberculous* (n=5), *non-tuberculous* (n=2), fungal (n=8), viral (n=1), *Chlamydia* (n=5), and mixed (n=4, 2 were infected with bacterium and fungus, and 2 were infected with bacterium and viruses) types (**Figure 5A**). Among 15 patients diagnosed with bacterial infection, the major pathogens were cultured positive in 3 cases (20%), and were detected in 7 (46.7%) and 6 cases (40%) respectively by Illumina and Nanopore. With the clinical etiological diagnosis as the reference standard, contingency tables for the Illumina and Nanopore in detecting different classes of pathogenic microorganisms were shown in **Figure 5B**. In terms of diagnostic value, the diagnostic area under ROC curve of the Nanopore was higher than that of Illumina in fungus (0.81 versus 0.73), but lower than that of Illumina in bacteria and chlamydia psittaci (0.60 versus 0.73, 0.8 versus 1.0, respectively) (**Figure 5C**).

### The guidance value of the three methods for treatment

**Figure 6:**
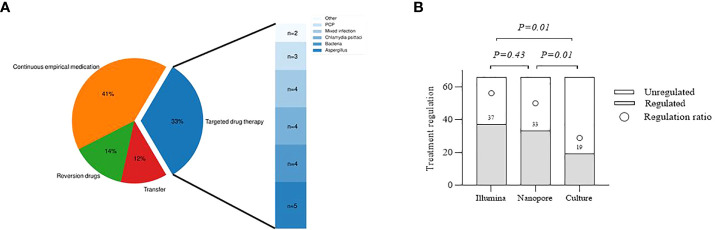
Evaluation of the clinical utility of the three methods. **(A)**, Distribution of antibiotic therapy and pathogens for targeted drug use. Continuous empirical medication, including unnecessary drug use for a specific pathogen and empiric treatment for an undefined pathogen. Transfer, Infectious pathogens detected should be treated at designated hospitals. Other, Drug modification according to other primary diseases. **(B)**, Comparison of different methods to guide the treatment.

Distribution of antibiotic therapy and pathogens for targeted drug use are shown in **Figure 6A**. Eight patients were transferred to other hospitals for further treatment, among whom 5 were diagnosed with tuberculosis infection, 2 with NTM infection, and 1 with unknown infection. Twenty-seven patients were treated with experiential therapy, including those with previously administered antibiotics related to the diagnosed pathogens and those without definite pathogens detected. Besides, antibiotics used in 9 patients were degraded based on pathogenic testing results. In 22 patients receiving targeted drug modification, bacterial infection was diagnosed in 4 patients, fungal infection in 8 patients, chlamydia psittaci infection in 4 patients and mixed infection in 4 patients, and the remaining 2 were for primary disease. With respect to the guidance value of the three methods for treatment, it was observed no statistically significant difference between Illumina and Nanopore (56.1% vs. 50%, *p*=0.43), but both were superior to the culture (56.1% vs. 28.8%, *p*=0.01; 50% vs. 28.8%, *p*=0.01) (**Figure 6B**).

### Levels of inflammatory indicators in culture-negative BALF

**Figure 7:**
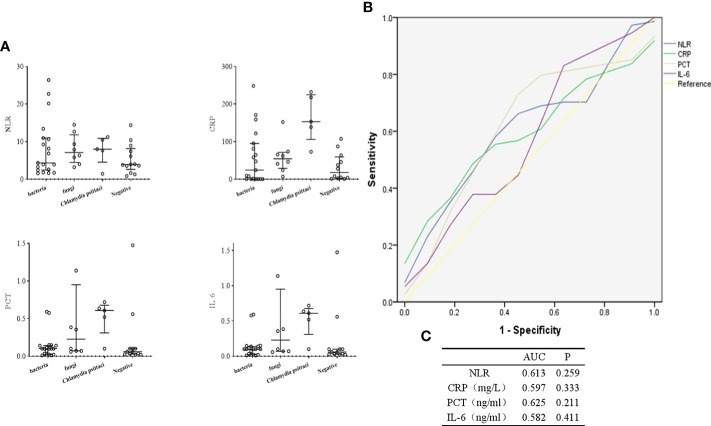
Diagnostic value of inflammatory indicators in culture-negative BALF of suspected CAP patients. **(A)**, Comparison of inflammatory markers in different types of pathogenic infection. **(B, C)**, ROC curves for inflammatory indicators. NLR, Neutrophil to lymphocyte count ratio.

The levels of inflammatory indicators in different types of pathogenic infection were compared in **Figure 7A**. The C-reactive protein (CRP) level of patients infected with *Chlamydia psittaci* was higher than those without. The procalcitonin (PCT) and interleukin (IL) 6 levels of patients with fungal and *Chlamydia psittaci* infection were higher than those without. Furthermore, we evaluated the efficacy of these inflammatory markers in diagnosing infection. As shown in **Figures 7B, C**, CRP, PCT or IL-6 alone could not be used to determine whether the patients with culture-negative BALF had infection.

### Evaluation on the timeliness of Nanopore technology

**Figure 8:**
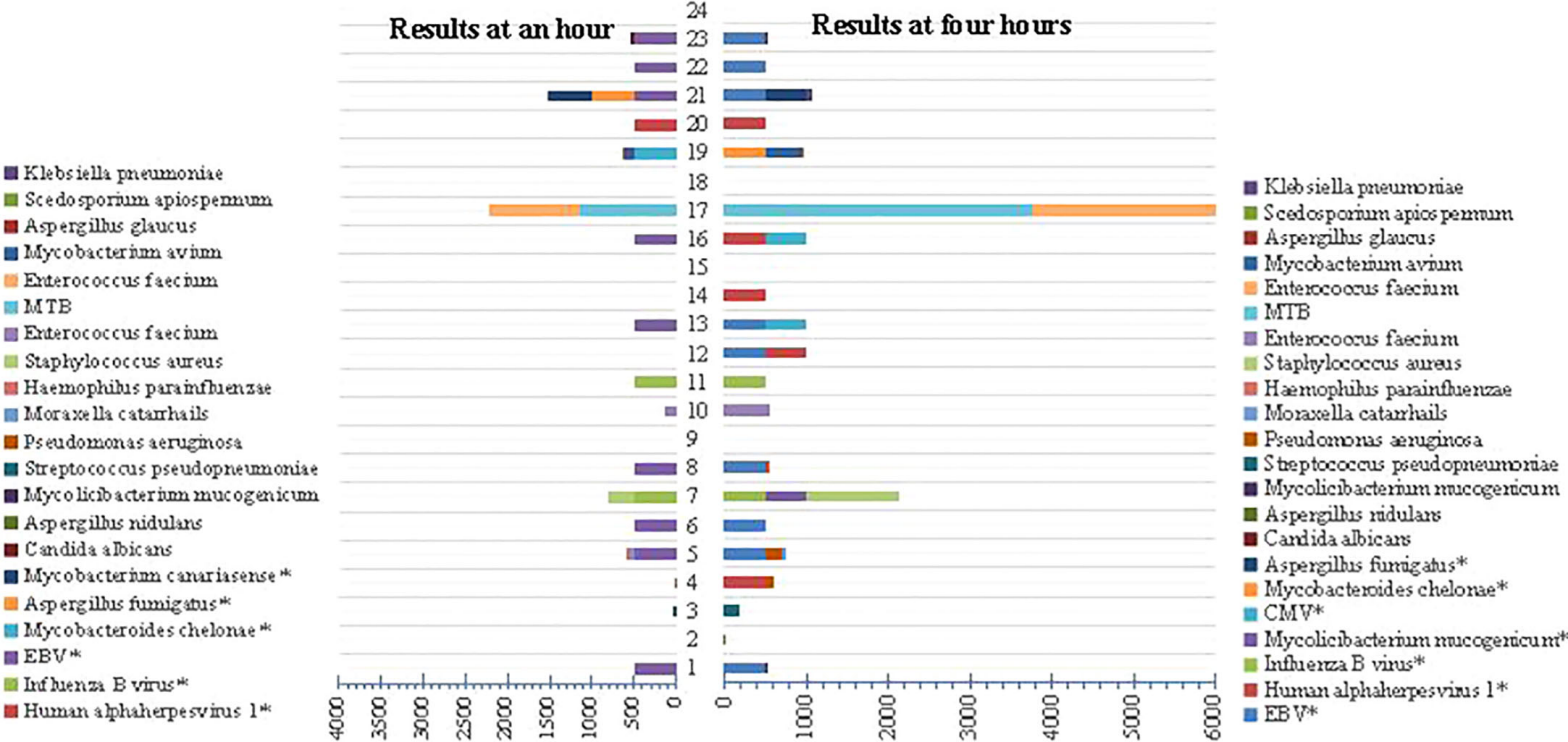
Comparison of genus distribution at an hour and 4 hours using Nanopore technology. “*” represents targeted sequencing detection results.

The genus distribution of Nanopore technology was observed respectively at an hour and four hours in **Figure 8**. It could be found that the species detected at an hour and four hours through Nanopore were consistent to some extent, and the TAT was significantly shorter than that by Illumina (14 h (11, 15) vs. 20 h (19, 21), *p*<0.01).

## Discussion

In terms of pathogenic detectability, we deduced that Nanopore and Illumina both had its own advantages in the identification of the main pathogens in BALF of CAP patients, including Illumina in the diagnosis of bacterium and Chlamydia psittaci and Nanopore in the detection of fungus. In the diagnosis of infection and guidance of antibiotic treatment, however, there was no significant difference between the two platforms, and both were superior to the culture method. Notably, Nanopore had a relatively short TAT, which may be promising in rapid etiological diagnosis of acute and critically ill patients.

In a large-scale prospective study, the pathogens in 63 out of 161 patients with bacterial infection of the lower respiratory tract were identified using rapid metagenomics with the sensitivity of 96.6% and specificity of 88.0%, indicating rapid metagenomic sequencing improved pathogenic detection in lower respiratory infection. Empirical antimicrobial therapy could be de-escalated if rapid metagenomic sequencing was hypothetically applied to clinical management (Mu et al., 2021). In our study, 80.3% of infection was established or excluded by Illumina and Nanopore, which was promising for clinical diagnosis. In terms of diagnostic performance for the major pathogens, Nanopore was superior to Illumina in fungus, but inferior to Illumina in bacteria and Chlamydia psittaci. Notably, among the 13 patients ultimately excluded from infection, 4 and 9 positive cases were detected by Illumina and Nanopore, respectively. In addition, there were still some results not accepted clinically or inconsistent with the final diagnosis. Therefore, the interpretation of clinical reports remains a challenge for metagenomic application. Inflammatory indicators provide an important basis for judging infection at present. We retrospectively analyzed common laboratory indicators of 40 confirmed infections and 13 excluded infections in this study. Due to the small amount of data, statistical analysis was not conducted. When evaluating the diagnostic value of a single inflammatory indicator in the positive and negative groups, the overall area under the ROC curve was modest and had no statistical significance. Therefore, we conclude that the diagnostic value of a single inflammatory indicator in patients with suspected CAP was limited. On the other hand, metagenomic sequencing-based pathogenic diagnostic methods have potential clinical application value. Therefore, it is necessary to promote the clinical application of metagenomic sequencing platform to further improve the efficiency of clinical diagnosis and treatment of pulmonary infection patients.

In this study, more types of species were detected by Nanopore than Illumina, especially in viruses, fungus and mycobacterium. Illumina and Nanopore were similar in the detection of bacterium, and both detected a type of Chlamydia. Nanopore applied the dehosting process in the sample pre-processing process, while Illumina did not carry out the dehosting process and directly carried out nucleic acid extraction. Moreover, the Nanopore platform applied the targeted enrichment method to improve the detection rate of DNA and RNA viruses. This may be a reason for the impact of microbial detection on the two different platforms. In accordance with previous reports (Liu et al., 2022), *Candida Albicans* was the most detected fungi whether by Illumina or Nanopore. The most frequently detected viruses included *EBV*, *CMV* and *Human alphaherpesvirus 1*, which were considered no pathogenic significance when detected in BALF in most studies. However, they might be pathogenic when the patient’s immune function was low. The number of patients with EBV detected by Nanopore were particularly high, which might be associated with the high carrying rate of the population. Although the value of some viruses detected by Nanopore is limited for CAP, it is still worthy of further study for the individual health management.

Some scholars compared the diagnostic efficiency of Illumina and Nanopore in various samples based on the known pathogens detected by the gold standard method as the reference standard, and found that the relative advantages of Illumina in bacteria and the relative advantages of Nanopore in fungus (Gu et al., 2021). In addition, we compared the detection efficiency of atypical pathogens such as Chlamydia psittaci on this basis, and found that Illumina detected all pathogens in BALF of 5 patients with chlamydia psittaci infection, while Nanopore detected three of them. The possible reason is that Nanopore includes metagenomics sequencing and targeted gene testing, while chlamydia psitsiti is not within the scope of targeted gene testing. Among the 40 patients diagnosed with CAP, 5 were clinically diagnosed with chlamydia psittaci infection, which was similar to that reported by others (Wang et al., 2021). With the popularization and application of metagenomics, the positive rate of chlamydia psittaci is also increasing, which needs to be paid attention to clinically. In addition, there were 5 cases of pneumocystis jiroveci infection, among which 4 cases were detected by Illumina, and all were detected by Nanopore, with a sensitivity and specificity of 75%, 100% by Illumina, 100% and 100% by Nanopore, respectively. Sun (Sun et al., 2022) reported the metagenomic diagnostic value of BALF infected with pneumocystis jiroveci in non-HIV immunosuppressed patients, and the results were not significantly different from this study.

Yang et al. (2022) believed that mNGS was helpful for the treatment of severe hospital-acquired pneumonia. Zinter et al. (2019) used mNGS to detect the pathogens in 41 lower respiratory tract samples of immunocompromised children, and found that mNGS could increase the sensitivity of clinical detection for missed pulmonary pathogens. In this study, the coincidence rates of infection diagnosis between Illumina and Nanopore with etiological diagnosis were 56.1% and 57.6%, respectively, which were much higher than the culture method (25%). Inevitably, in combination with laboratory tests, 5 were identified with infection of unknown causes, 6 were probable infection, and 2 were infected due to other non-infectious factors like lung damage and cancer. Empiric antibiotic therapy is still the main treatment for this population. Furthermore, TAT detected by Illumina and Nanopore was much shorter than that by the culture. Illumina reports the results within 24 h; Nanopore performs pre-analysis at 50 min and feedbacks the results at 6 h (Gu et al., 2021). In this study, the significant advantage of Nanopore was that it could obtain a pre-report within an hour to guide the clinical treatment timely.

Currently, there are guidelines for empiric treatment options for patients with multiple types of lung infection, which greatly improve the probability of initial treatment success. The patients were reported to benefit from the timely detection of pathogens by mNGS and most of them had better prognosis (Liu et al., 2022). In this study, 22 cases were adjusted for targeted drugs based on Illumina and Nanopore, among whom 4 showed culture-positive. It can be estimated that the rate of antibiotic use based on mNGS platforms was increased to 27.3%. Additionally, the patients with fungal, atypical pathogenic infection accounted for 54.5% of the patients adjusted for targeted drugs, but these pathogens were difficult to be detected by traditional methods. Liu et al. reported mNGS as an auxiliary method for the diagnosis of mucormycosis (Liu et al., 2021). Our results showed that Illumina-based and Nanopore-based mNGS was similar in guiding antibiotic therapy, superior to the culture method.

This study is a real-world application evaluation. We performed etiological examination of BALF in suspected CAP patients based on metagenomic sequencing technologies of the two platforms, and evaluated the clinical value of infection diagnosis, pathogen compliance and treatment guidance. Of course, due to the small population included in this study, there may be some selection bias in the results. Since only 3 cases were (Including two mixed infection) diagnosed as viral infection in this study, the diagnostic value of the three methods for viral infection was not evaluated. Due to the small sample size of inflammatory indicators in culture-negative BALF, no further statistical analysis was conducted. In addition, we did not further analyze the genes associated with drug resistance, which definitely aid the diagnosis and consideration of antibiotics. We will further conduct large-sample, multi-center, multi-dimensional, prospective clinical studies in the near future to more clearly evaluate the clinical value of the two platforms for infection diagnosis, treatment and prognosis.

## Conclusions

Our results suggest that Illumina and Nanopore both have its own advantages for the detection of pathogens in BALF of suspected CAP patients, and play similar roles in infection diagnosis and guiding clinical treatment. Additionally, Nanopore presents a relatively short TAT, which may be promising in rapid etiological diagnosis of acute and critically ill patients.

## Data availability statement

The datasets presented in this study can be found in online repositories. The names of the repository/repositories and accession number(s) can be found at https://ngdc.cncb.ac.cn/omix/, OMIX006862.

## Ethics statement

This study involving human participants was reviewed and approved by Ethics. Committee of Nanjing First Hospital (NO. KF20220516-05).

## Author contributions

JZ, LG, CZ participated in writing the manuscript. CS, HD, YT and LW conduct the study design. ZW, YC, ZY provide clinical information and case data. ZL, JJ take part in a discussion and analysis of data. All authors contributed to the article and approved the submitted version.

## Funding

This work was supported by National Natural Science Foundation of China (No.: 82100095).

## Acknowledgments

We thank Furong Du, Xiaotong Xi for helpful discussions from Nanjing Simcere Diagnostics Co., Ltd.

## Conflict of interest

Author CZ, CS, HD, ZL were employed by Jiangsu Simcere Diagnostics Co.

The remaining authors declare that the research was conducted in the absence of any commercial or financial relationships that could be construed as a potential conflict of interest.

## Publisher’s note

All claims expressed in this article are solely those of the authors and do not necessarily represent those of their affiliated organizations, or those of the publisher, the editors and the reviewers. Any product that may be evaluated in this article, or claim that may be made by its manufacturer, is not guaranteed or endorsed by the publisher.
