# Corrigendum: Clinical value of metagenomic next-generation sequencing by Illumina and Nanopore for the detection of pathogens in bronchoalveolar lavage fluid in suspected community-acquired pneumonia patients

**DOI:** 10.3389/fcimb.2024.1468511

**Published:** 2024-08-01

**Authors:** Jing Zhang, Lin Gao, Chi Zhu, Jiajia Jin, Chao Song, Hang Dong, Zhenzhong Li, Zheng Wang, Yubao Chen, Zhenhua Yang, Yan Tan, Li Wang

**Affiliations:** ^1^ Department of Respiratory and Critical Care Medicine, Nanjing First Hospital, Nanjing Medical University, Nanjing, China; ^2^ State Key Laboratory of Translational Medicine and Innovative Drug Development, Jiangsu Simcere Diagnostics Co., Ltd., Nanjing, China

**Keywords:** metagenomic next-generation sequencing, bronchoalveolar lavage fluid, community-acquired pneumonia, Illumina, nanopore, diagnostic value, pathogenic identification

## Incorrect data availability statement

In the published article, there was an error in the Data Availability statement. The original Data Availability statement: The original contributions presented in the study are included in the article/supplementary material. Further inquiries can be directed to the corresponding authors.

The correct Data Availability statement appears below.

## Data availability statement

The datasets presented in this study can be found in online repositories. The names of the repository/repositories and accession number(s) can be found at https://ngdc.cncb.ac.cn/omix/, OMIX006862.

The authors apologize for this error and state that this does not change the scientific conclusions of the article in any way. The original article has been updated.

